# Pulsatile blood flow, shear force, energy dissipation and Murray's Law

**DOI:** 10.1186/1742-4682-3-31

**Published:** 2006-08-21

**Authors:** Page R Painter, Patrik Edén, Hans-Uno Bengtsson

**Affiliations:** 1 Office of Environmental Health Hazard Assessment, California Environmental Protection Agency, P. O. Box 4010, Sacramento, California 95812, USA; 2 Department of Theoretical Physics, Lund University, S-223 62 Soelvegatan 14A, Lund, Sweden

## Abstract

**Background:**

Murray's Law states that, when a parent blood vessel branches into daughter vessels, the cube of the radius of the parent vessel is equal to the sum of the cubes of the radii of daughter blood vessels. Murray derived this law by defining a cost function that is the sum of the energy cost of the blood in a vessel and the energy cost of pumping blood through the vessel. The cost is minimized when vessel radii are consistent with Murray's Law. This law has also been derived from the hypothesis that the shear force of moving blood on the inner walls of vessels is constant throughout the vascular system. However, this derivation, like Murray's earlier derivation, is based on the assumption of constant blood flow.

**Methods:**

To determine the implications of the constant shear force hypothesis and to extend Murray's energy cost minimization to the pulsatile arterial system, a model of pulsatile flow in an elastic tube is analyzed. A new and exact solution for flow velocity, blood flow rate and shear force is derived.

**Results:**

For medium and small arteries with pulsatile flow, Murray's energy minimization leads to Murray's Law. Furthermore, the hypothesis that the maximum shear force during the cycle of pulsatile flow is constant throughout the arterial system implies that Murray's Law is approximately true. The approximation is good for all but the largest vessels (aorta and its major branches) of the arterial system.

**Conclusion:**

A cellular mechanism that senses shear force at the inner wall of a blood vessel and triggers remodeling that increases the circumference of the wall when a shear force threshold is exceeded would result in the observed scaling of vessel radii described by Murray's Law.

## Background

In 1926, the physiologist Cecil Murray published a theoretical explanation for the relationship between the radius of an artery immediately upstream from a branch point (parent artery) and the radii of arteries immediately downstream (daughter arteries) [1,2]. In its simplest form, i.e., when an artery of radius *R*_*k *_branches into *η *arteries of radius *R*_*k*+1_, the relationship termed Murray's Law states that Rk3=ηRk+13
 MathType@MTEF@5@5@+=feaafiart1ev1aaatCvAUfKttLearuWrP9MDH5MBPbIqV92AaeXatLxBI9gBaebbnrfifHhDYfgasaacH8akY=wiFfYdH8Gipec8Eeeu0xXdbba9frFj0=OqFfea0dXdd9vqai=hGuQ8kuc9pgc9s8qqaq=dirpe0xb9q8qiLsFr0=vr0=vr0dc8meaabaqaciaacaGaaeqabaqabeGadaaakeaacqWGsbGudaqhaaWcbaGaem4AaSgabaGaeG4mamdaaOGaeyypa0dcciGae83TdGMaemOuai1aa0baaSqaaiabdUgaRjabgUcaRiabigdaXaqaaiabiodaZaaaaaa@389B@. Murray's derivation of this relationship assumed that there is an energy requirement for producing the blood contained in a vessel that is proportional to the volume of blood in a vessel and that there is an energy requirement for pumping blood through a vessel that is given by Poiseuille's Law for flow in a tube. When the radius of a branching artery is increased, the cost of blood in the artery increases, but the cost of pumping blood through the artery decreases. Calculation of the radius that minimizes cost, using the calculus of variations, leads to Murray's Law.

While Murray did not suggest a mechanism for the regulation of the radius of an artery, other scientists have. A recent hypothesis is that shear force at the inner surface triggers circumferential growth if the force is above a threshold [3,4]. This is an attractive hypothesis because high values of shear in fluids can damage or destroy cells. Furthermore, turbulence, which occurs above critical values of shear in fluids, is associated with atherosclerosis of arterial walls.

Implications of constant shear force at the wall of blood vessels have been analyzed by Kassab and Fung for a constant pressure gradient model [5]. They showed that, if shear force at the inner wall of vessels is constant in their model, then scaling of the radius of blood vessels is described by Murray's Law.

If the shear-force remodeling (SFR) hypothesis is correct, it should be possible to derive Murray's Law from the equations describing the fluid mechanics of pulsatile blood flow in a tubular structure. Specifically, it should be possible to derive the law from the basic work of Womersley on pulsatile flow in an elastic tube [6]. However, the results of Womersley are complex, and the approximations introduced by Womersley may result in inaccurate predictions. Furthermore, the elastic tube in Womersley's model is not tethered to surrounding structures. Therefore, we analyze an elastic tube model where the inside surface can move relative to the outside surface but where the outside wall is attached to surrounding structures. We also use a general solution for the differential equation describing the pulsatile flow model that was not used directly by Womersley and that leads to an exact solution. We show that the exact solution can be closely approximated by a simpler expression and use this result to analyze the relationship between vessel radius and shear force during pulsatile blood flow.

### The rigid tube model

To develop the model, we first consider blood flow in a rigid cylindrical tube of constant radius *R*. We make the same assumptions as Kassab and Fung: that "blood is an incompressible viscous fluid so that flow is described by the Navier-Stokes equation" and that "each blood vessel is a straight circular cylindrical tube" [5]. The distance from the central axis of the tube is denoted *r*, the tube length is *L*, and the velocity of flow is *u*. The Navier-Stokes equation of motion is

μ∂2u∂r2+μ1r∂u∂r+A=ρ∂u∂t,
 MathType@MTEF@5@5@+=feaafiart1ev1aaatCvAUfKttLearuWrP9MDH5MBPbIqV92AaeXatLxBI9gBaebbnrfifHhDYfgasaacH8akY=wiFfYdH8Gipec8Eeeu0xXdbba9frFj0=OqFfea0dXdd9vqai=hGuQ8kuc9pgc9s8qqaq=dirpe0xb9q8qiLsFr0=vr0=vr0dc8meaabaqaciaacaGaaeqabaqabeGadaaakeaaiiGacqWF8oqBdaWcaaqaaiabgkGi2oaaCaaaleqabaGaeGOmaidaaOGaemyDauhabaGaeyOaIyRaemOCai3aaWbaaSqabeaacqaIYaGmaaaaaOGaey4kaSIae8hVd02aaSaaaeaacqaIXaqmaeaacqWGYbGCaaWaaSaaaeaacqGHciITcqWG1bqDaeaacqGHciITcqWGYbGCaaGaey4kaSIaemyqaeKaeyypa0Jae8xWdi3aaSaaaeaacqGHciITcqWG1bqDaeaacqGHciITcqWG0baDaaGaeiilaWcaaa@4C81@

where *A *is the pressure gradient Δ*P/L*, *μ *is viscosity and *ρ *is density. Substituting *ξ *for *μ*/*ρ *gives

ξ∂2u∂r2+ξ1r∂u∂r+Aρ=∂u∂t.     (1)
 MathType@MTEF@5@5@+=feaafiart1ev1aaatCvAUfKttLearuWrP9MDH5MBPbIqV92AaeXatLxBI9gBaebbnrfifHhDYfgasaacH8akY=wiFfYdH8Gipec8Eeeu0xXdbba9frFj0=OqFfea0dXdd9vqai=hGuQ8kuc9pgc9s8qqaq=dirpe0xb9q8qiLsFr0=vr0=vr0dc8meaabaqaciaacaGaaeqabaqabeGadaaakeaaiiGacqWF+oaEdaWcaaqaaiabgkGi2oaaCaaaleqabaGaeGOmaidaaOGaemyDauhabaGaeyOaIyRaemOCai3aaWbaaSqabeaacqaIYaGmaaaaaOGaey4kaSIae8NVdG3aaSaaaeaacqaIXaqmaeaacqWGYbGCaaWaaSaaaeaacqGHciITcqWG1bqDaeaacqGHciITcqWGYbGCaaGaey4kaSYaaSaaaeaacqWGbbqqaeaacqWFbpGCaaGaeyypa0ZaaSaaaeaacqGHciITcqWG1bqDaeaacqGHciITcqWG0baDaaGaeiOla4IaaCzcaiaaxMaadaqadiqaaiabigdaXaGaayjkaiaawMcaaaaa@506E@

Let *ũ *be the initial-condition-independent solution of Equation (1) when the pressure gradient is a constant (denoted *Ã*). The solution is Poiseuille's equation

u˜=A˜(R2−r2)/(4μ).
 MathType@MTEF@5@5@+=feaafiart1ev1aaatCvAUfKttLearuWrP9MDH5MBPbIqV92AaeXatLxBI9gBaebbnrfifHhDYfgasaacH8akY=wiFfYdH8Gipec8Eeeu0xXdbba9frFj0=OqFfea0dXdd9vqai=hGuQ8kuc9pgc9s8qqaq=dirpe0xb9q8qiLsFr0=vr0=vr0dc8meaabaqaciaacaGaaeqabaqabeGadaaakeaacuWG1bqDgaacaiabg2da9iqbdgeabzaaiaWaaeWaceaacqWGsbGudaahaaWcbeqaaiabikdaYaaakiabgkHiTiabdkhaYnaaCaaaleqabaGaeGOmaidaaaGccaGLOaGaayzkaaGaei4la8YaaeWaceaacqaI0aaniiGacqWF8oqBaiaawIcacaGLPaaacqGGUaGlaaa@3DBA@

The rate of blood flow in the tube, Q˜
 MathType@MTEF@5@5@+=feaafiart1ev1aaatCvAUfKttLearuWrP9MDH5MBPbIqV92AaeXatLxBI9gBaebbnrfifHhDYfgasaacH8akY=wiFfYdH8Gipec8Eeeu0xXdbba9frFj0=OqFfea0dXdd9vqai=hGuQ8kuc9pgc9s8qqaq=dirpe0xb9q8qiLsFr0=vr0=vr0dc8meaabaqaciaacaGaaeqabaqabeGadaaakeaacuWGrbqugaacaaaa@2DE6@, is

∫0R2πru˜dr=πA˜R4/(8μ),     (3)
 MathType@MTEF@5@5@+=feaafiart1ev1aaatCvAUfKttLearuWrP9MDH5MBPbIqV92AaeXatLxBI9gBaebbnrfifHhDYfgasaacH8akY=wiFfYdH8Gipec8Eeeu0xXdbba9frFj0=OqFfea0dXdd9vqai=hGuQ8kuc9pgc9s8qqaq=dirpe0xb9q8qiLsFr0=vr0=vr0dc8meaabaqaciaacaGaaeqabaqabeGadaaakeaadaWdXaqaaiabikdaYGGaciab=b8aWjabdkhaYjqbdwha1zaaiaGaemizaqMaemOCaiNaeyypa0Jae8hWdaNafmyqaeKbaGaacqWGsbGudaahaaWcbeqaaiabisda0aaakiabc+caVmaabmGabaGaeGioaGJae8hVd0gacaGLOaGaayzkaaaaleaacqaIWaamaeaacqWGsbGua0Gaey4kIipakiabcYcaSiaaxMaacaWLjaWaaeWaceaacqaIZaWmaiaawIcacaGLPaaaaaa@4952@

and the shear force (per unit area) of the fluid on the inner wall of the tube is

−μ[∂u˜/∂r|r=RA˜R/2.     (4)
 MathType@MTEF@5@5@+=feaafiart1ev1aaatCvAUfKttLearuWrP9MDH5MBPbIqV92AaeXatLxBI9gBaebbnrfifHhDYfgasaacH8akY=wiFfYdH8Gipec8Eeeu0xXdbba9frFj0=OqFfea0dXdd9vqai=hGuQ8kuc9pgc9s8qqaq=dirpe0xb9q8qiLsFr0=vr0=vr0dc8meaabaqaciaacaGaaeqabaqabeGadaaakeaacqGHsisliiGacqWF8oqBdaabciqaaiabcUfaBjabgkGi2kqbdwha1zaaiaGaei4la8IaeyOaIyRaemOCaihacaGLiWoadaWgaaWcbaGaemOCaiNaeyypa0JaemOuaifabeaakiqbdgeabzaaiaGaemOuaiLaei4la8IaeGOmaiJaeiOla4IaaCzcaiaaxMaadaqadiqaaiabisda0aGaayjkaiaawMcaaaaa@456B@

Now consider the rigid tube model with an oscillating pressure gradient *Ăe*^*iωt*^. The solution for the velocity, *ŭ*, stated by Womersley [6], is

*ŭ *= [*Ă*/(*ρiω*)]*e*^*iωt*^[1 - *J*_0_(*i*^3/2^*αr*)/*J*_0_(*i*^3/2^*αR*)],

where *α *= (*ω*/*ξ*)^1/2 ^and *J*_0_(*i*^3/2^*αr*) is the Bessel function of order 0,

∑n=0n=∞(−1)n(i3/2αr/2)2n/(n!n!).
 MathType@MTEF@5@5@+=feaafiart1ev1aaatCvAUfKttLearuWrP9MDH5MBPbIqV92AaeXatLxBI9gBaebbnrfifHhDYfgasaacH8akY=wiFfYdH8Gipec8Eeeu0xXdbba9frFj0=OqFfea0dXdd9vqai=hGuQ8kuc9pgc9s8qqaq=dirpe0xb9q8qiLsFr0=vr0=vr0dc8meaabaqaciaacaGaaeqabaqabeGadaaakeaadaaeWaqaamaabmGabaGaeyOeI0IaeGymaedacaGLOaGaayzkaaWaaWbaaSqabeaacqWGUbGBaaGcdaqadiqaaiabdMgaPnaaCaaaleqabaGaeG4mamJaei4la8IaeGOmaidaaGGacOGae8xSdeMaemOCaiNaei4la8IaeGOmaidacaGLOaGaayzkaaaaleaacqWGUbGBcqGH9aqpcqaIWaamaeaacqWGUbGBcqGH9aqpcqGHEisPa0GaeyyeIuoakmaaCaaaleqabaGaeGOmaiJaemOBa4gaaOGaei4la8YaaeWaceaacqWGUbGBcqGGHaqicqWGUbGBcqGGHaqiaiaawIcacaGLPaaacqGGUaGlaaa@5006@

Womersley did not provide a derivation of the solution for the rigid tube. He cited Lambossy [7] as the source, but Lambossy did not arrive at this solution for *ŭ*. Therefore, we provide a derivation of the solution for the rigid tube model that can be extended to a solution for an elastic tube model. We write *ŭ *as a power series of the variable *r*:

*ŭ *= *b*_0 _+ *b*_1_*r *+ *b*_2_*r*^2 ^+ ...     (5)

where *b*_*i *_is a function of time, *t*. Equating the coefficients of *r*^0 ^following substitution of this power series into Equation (1) gives

2ξb2+2ξb2+A⌣eiωt/ρ=b0/     (6)
 MathType@MTEF@5@5@+=feaafiart1ev1aaatCvAUfKttLearuWrP9MDH5MBPbIqV92AaeXatLxBI9gBaebbnrfifHhDYfgasaacH8akY=wiFfYdH8Gipec8Eeeu0xXdbba9frFj0=OqFfea0dXdd9vqai=hGuQ8kuc9pgc9s8qqaq=dirpe0xb9q8qiLsFr0=vr0=vr0dc8meaabaqaciaacaGaaeqabaqabeGadaaakeaacqaIYaGmiiGacqWF+oaEcqWGIbGydaWgaaWcbaGaeGOmaidabeaakiabgUcaRiabikdaYiab=57a4jabdkgaInaaBaaaleaacqaIYaGmaeqaaOGaey4kaSIafmyqaeKbaqbacqWGLbqzdaahaaWcbeqaaiabdMgaPjab=L8a3jabdsha0baakiabc+caViab=f8aYjabg2da9iabdkgaInaaDaaaleaacqaIWaamaeaacqGGVaWlaaGccaWLjaGaaCzcamaabmGabaGaeGOnaydacaGLOaGaayzkaaaaaa@4AD2@

where bn/
 MathType@MTEF@5@5@+=feaafiart1ev1aaatCvAUfKttLearuWrP9MDH5MBPbIqV92AaeXatLxBI9gBaebbnrfifHhDYfgasaacH8akY=wiFfYdH8Gipec8Eeeu0xXdbba9frFj0=OqFfea0dXdd9vqai=hGuQ8kuc9pgc9s8qqaq=dirpe0xb9q8qiLsFr0=vr0=vr0dc8meaabaqaciaacaGaaeqabaqabeGadaaakeaacqWGIbGydaqhaaWcbaGaemOBa4gabaGaei4la8caaaaa@3071@ = *db*_*n*_/*dt*. Equating the coefficients of *r*^*n *^for *n*>*0 *gives

(n+2)(n+1)ξbn+2+(n+2)ξbn+2=bn/.
 MathType@MTEF@5@5@+=feaafiart1ev1aaatCvAUfKttLearuWrP9MDH5MBPbIqV92AaeXatLxBI9gBaebbnrfifHhDYfgasaacH8akY=wiFfYdH8Gipec8Eeeu0xXdbba9frFj0=OqFfea0dXdd9vqai=hGuQ8kuc9pgc9s8qqaq=dirpe0xb9q8qiLsFr0=vr0=vr0dc8meaabaqaciaacaGaaeqabaqabeGadaaakeaadaqadiqaaiabd6gaUjabgUcaRiabikdaYaGaayjkaiaawMcaamaabmGabaGaemOBa4Maey4kaSIaeGymaedacaGLOaGaayzkaaacciGae8NVdGNaemOyai2aaSbaaSqaaiabd6gaUjabgUcaRiabikdaYaqabaGccqGHRaWkdaqadiqaaiabd6gaUjabgUcaRiabikdaYaGaayjkaiaawMcaaiab=57a4jabdkgaInaaBaaaleaacqWGUbGBcqGHRaWkcqaIYaGmaeqaaOGaeyypa0JaemOyai2aa0baaSqaaiabd6gaUbqaaiabc+caVaaakiabc6caUaaa@4E91@

Solving for *b*_*n*+2 _gives

bn+2=(1/ξ)bn//(n+2)2.     (7)
 MathType@MTEF@5@5@+=feaafiart1ev1aaatCvAUfKttLearuWrP9MDH5MBPbIqV92AaeXatLxBI9gBaebbnrfifHhDYfgasaacH8akY=wiFfYdH8Gipec8Eeeu0xXdbba9frFj0=OqFfea0dXdd9vqai=hGuQ8kuc9pgc9s8qqaq=dirpe0xb9q8qiLsFr0=vr0=vr0dc8meaabaqaciaacaGaaeqabaqabeGadaaakeaacqWGIbGydaWgaaWcbaGaemOBa4Maey4kaSIaeGOmaidabeaakiabg2da9maabmGabaGaeGymaeJaei4la8ccciGae8NVdGhacaGLOaGaayzkaaGaemOyai2aa0baaSqaaiabd6gaUbqaaiabc+caVaaakiabc+caVmaabmGabaGaemOBa4Maey4kaSIaeGOmaidacaGLOaGaayzkaaWaaWbaaSqabeaacqaIYaGmaaGccqGGUaGlcaWLjaGaaCzcamaabmGabaGaeG4naCdacaGLOaGaayzkaaaaaa@46EA@

Because *∂ŭ*/*∂r *= 0 at *r *= 0, *b*_1 _is 0 for all values of *t*. Consequently, for all odd values of *n*, *b*_*n *_is 0.

We now define the constant *B*_1,0 _by the equation

*b*_2 _= [(*iω*/*ξ*)/4]*e*^*iωt*^*B*_1,0_.     (8)

From this equation and Equations (5) and (7) it follows that the series

*b*_2_*r*^2 ^+ *b*_4_*r*^4 ^+ *b*_6_*r*^6 ^+ ... is

*B*_1,0_*e*^*iωt *^{[(*iωr*^2^/ξ)/4]/(1!)^2 ^+ [(*iωr*^2^/*ξ*)/4]^2^/(2!)^2 ^+ [(*iωr*^2^/*ξ*)/4]^3^/(3!)^2 ^+ ...}.

From Equation (6) and Equation (8), it follows that

*b*_0 _= [*Ă*/(*ρiω*)]*e*^*iωt *^+ *B*_1,0_*e*^*iωt*^.

Consequently, the expression for blood velocity is

*ŭ *= [*Ă*/(*ρiω*)]*e*^*iωt *^+ *B*_1,0_*e*^*iωt *^*J*_0_(*i*^3/2^*αr*).     (9)

The above derivation is similar to many published analyses of differential equations that have solutions containing Bessel functions. The derivation is provided to make it clear that all solutions of the model of blood flow in a rigid tube in response to the pressure gradient *Ăe*^*iωt *^are described by the above expression. This solution can also be derived from the assumption that we can write *ŭ *= *νe*^*iωt*^, where *ν *is a function of the single variable *r*. Substitution into Equation (1) leads to

ξ∂2ν∂r2+ξ1r∂ν∂r+A⌣ρ=iων,
 MathType@MTEF@5@5@+=feaafiart1ev1aaatCvAUfKttLearuWrP9MDH5MBPbIqV92AaeXatLxBI9gBaebbnrfifHhDYfgasaacH8akY=wiFfYdH8Gipec8Eeeu0xXdbba9frFj0=OqFfea0dXdd9vqai=hGuQ8kuc9pgc9s8qqaq=dirpe0xb9q8qiLsFr0=vr0=vr0dc8meaabaqaciaacaGaaeqabaqabeGadaaakeaaiiGacqWF+oaEdaWcaaqaaiabgkGi2oaaCaaaleqabaGaeeOmaidaaOGae8xVd4gabaGaeyOaIyRaemOCai3aaWbaaSqabeaacqaIYaGmaaaaaOGaey4kaSIae8NVdG3aaSaaaeaacqaIXaqmaeaacqWGYbGCaaWaaSaaaeaacqGHciITcqWF9oGBaeaacqGHciITcqWGYbGCaaGaey4kaSYaaSaaaeaacuWGbbqqgaafaaqaaiab=f8aYbaacqGH9aqpcqWGPbqAcqWFjpWDcqWF9oGBcqGGSaalaaa@4C55@

which can be written as

∂2ν∂r2+1r∂ν∂r+i3α2ν=−A⌣μ.
 MathType@MTEF@5@5@+=feaafiart1ev1aaatCvAUfKttLearuWrP9MDH5MBPbIqV92AaeXatLxBI9gBaebbnrfifHhDYfgasaacH8akY=wiFfYdH8Gipec8Eeeu0xXdbba9frFj0=OqFfea0dXdd9vqai=hGuQ8kuc9pgc9s8qqaq=dirpe0xb9q8qiLsFr0=vr0=vr0dc8meaabaqaciaacaGaaeqabaqabeGadaaakeaadaWcaaqaaiabgkGi2oaaCaaaleqabaGaeGOmaidaaGGacOGae8xVd4gabaGaeyOaIyRaemOCai3aaWbaaSqabeaacqaIYaGmaaaaaOGaey4kaSYaaSaaaeaacqaIXaqmaeaacqWGYbGCaaWaaSaaaeaacqGHciITcqWF9oGBaeaacqGHciITcqWGYbGCaaGaey4kaSIaemyAaK2aaWbaaSqabeaacqaIZaWmaaGccqWFXoqydaahaaWcbeqaaiabikdaYaaakiab=17aUjabg2da9iabgkHiTmaalaaabaGafmyqaeKbaqbaaeaacqWF8oqBaaGaeiOla4caaa@4BED@

We note that, if *ν*_0 _is a solution of the equation ∂2ν∂r2+1r∂ν∂r+i3α2ν=0
 MathType@MTEF@5@5@+=feaafiart1ev1aaatCvAUfKttLearuWrP9MDH5MBPbIqV92AaeXatLxBI9gBaebbnrfifHhDYfgasaacH8akY=wiFfYdH8Gipec8Eeeu0xXdbba9frFj0=OqFfea0dXdd9vqai=hGuQ8kuc9pgc9s8qqaq=dirpe0xb9q8qiLsFr0=vr0=vr0dc8meaabaqaciaacaGaaeqabaqabeGadaaakeaadaWcaaqaaiabgkGi2oaaCaaaleqabaGaeGOmaidaaGGacOGae8xVd4gabaGaeyOaIyRaemOCai3aaWbaaSqabeaacqaIYaGmaaaaaOGaey4kaSYaaSaaaeaacqaIXaqmaeaacqWGYbGCaaWaaSaaaeaacqGHciITcqWF9oGBaeaacqGHciITcqWGYbGCaaGaey4kaSIaemyAaK2aaWbaaSqabeaacqaIZaWmaaGccqWFXoqydaahaaWcbeqaaiabikdaYaaakiab=17aUjabg2da9iabicdaWaaa@4823@, which is Bessel's equation of order 0, then *ν*_0 _+ *A*/(*i α*^2 ^*μ*) is a solution of Equation (1) for the oscillating pressure gradient. Noting that the solution of Bessel's equation is *BJ*_0_(*i*^3/2^*αr*), where *B *is a constant, completes the derivation.

A boundary condition for the rigid tube is that the function *ŭ *in Equation (9) is 0 at *r *= *R*:

0 = [*Ă*/(*ρiω*)]*e*^*iωt *^+ *B*_1,0_*e*^*iωt *^*J*_0_(*i*^3/2^*αR*).

Consequently, *B*_1,0 _= -[*Ă*/(*ρiω*)]/*J*_0_(*i*^3/2^*αR*), and

*ŭ *= [*Ă*/(*ρiω*)]*e*^*iωt*^[1 - *J*_0_(*i*^3/2^*αr*)/*J*_0_(*i*^3/2^*αR*)],     (10)

which is the result published without derivation by Womersley [6].

The rate of blood flow Q⌣
 MathType@MTEF@5@5@+=feaafiart1ev1aaatCvAUfKttLearuWrP9MDH5MBPbIqV92AaeXatLxBI9gBaebbnrfifHhDYfgasaacH8akY=wiFfYdH8Gipec8Eeeu0xXdbba9frFj0=OqFfea0dXdd9vqai=hGuQ8kuc9pgc9s8qqaq=dirpe0xb9q8qiLsFr0=vr0=vr0dc8meaabaqaciaacaGaaeqabaqabeGadaaakeaacuWGrbqugaafaaaa@2DF2@ is computed as

∫0R2πru⌣dr=(πA⌣eiωt)/(iωρ)[R2−2R2J1(i3/2αR)/(i3/2αR)/J0(i3/2αR)],
 MathType@MTEF@5@5@+=feaafiart1ev1aaatCvAUfKttLearuWrP9MDH5MBPbIqV92AaeXatLxBI9gBaebbnrfifHhDYfgasaacH8akY=wiFfYdH8Gipec8Eeeu0xXdbba9frFj0=OqFfea0dXdd9vqai=hGuQ8kuc9pgc9s8qqaq=dirpe0xb9q8qiLsFr0=vr0=vr0dc8meaabaqaciaacaGaaeqabaqabeGadaaakeaadaWdXaqaaiabikdaYGGaciab=b8aWjabdkhaYjqbdwha1zaauaGaemizaqMaemOCaiNaeyypa0ZaaeWaceaacqWFapaCcuWGbbqqgaafaiabdwgaLnaaCaaaleqabaGaemyAaKMae8xYdCNaemiDaqhaaaGccaGLOaGaayzkaaaaleaacqaIWaamaeaacqWGsbGua0Gaey4kIipakiabc+caVmaabmGabaGaemyAaKMae8xYdCNae8xWdihacaGLOaGaayzkaaWaamWaceaacqWGsbGudaahaaWcbeqaaiabikdaYaaakiabgkHiTiabikdaYiabdkfasnaaCaaaleqabaGaeGOmaidaaOGaemOsaO0aaSbaaSqaaiabigdaXaqabaGcdaqadiqaaiabdMgaPnaaCaaaleqabaGaeG4mamJaei4la8IaeGOmaidaaOGae8xSdeMaemOuaifacaGLOaGaayzkaaGaei4la8YaaeWaceaacqWGPbqAdaahaaWcbeqaaiabiodaZiabc+caViabikdaYaaakiab=f7aHjabdkfasbGaayjkaiaawMcaaiabc+caViabdQeaknaaBaaaleaacqaIWaamaeqaaOWaaeWaceaacqWGPbqAdaahaaWcbeqaaiabiodaZiabc+caViabikdaYaaakiab=f7aHjabdkfasbGaayjkaiaawMcaaaGaay5waiaaw2faaiabcYcaSaaa@7604@

where *J*_1_(*i*^3/2^*αR*) is the Bessel function of order 1,

∑n=0n=∞(−1)n(i3/2αR/2)2n+1/[(n+1)!n!]
 MathType@MTEF@5@5@+=feaafiart1ev1aaatCvAUfKttLearuWrP9MDH5MBPbIqV92AaeXatLxBI9gBaebbnrfifHhDYfgasaacH8akY=wiFfYdH8Gipec8Eeeu0xXdbba9frFj0=OqFfea0dXdd9vqai=hGuQ8kuc9pgc9s8qqaq=dirpe0xb9q8qiLsFr0=vr0=vr0dc8meaabaqaciaacaGaaeqabaqabeGadaaakeaadaaeWaqaamaabmGabaGaeyOeI0IaeGymaedacaGLOaGaayzkaaWaaWbaaSqabeaacqWGUbGBaaaabaGaemOBa4Maeyypa0JaeGimaadabaGaemOBa4Maeyypa0JaeyOhIukaniabggHiLdGcdaqadiqaaiabdMgaPnaaCaaaleqabaGaeG4mamJaei4la8IaeGOmaidaaGGacOGae8xSdeMaemOuaiLaei4la8IaeGOmaidacaGLOaGaayzkaaWaaWbaaSqabeaacqaIYaGmcqWGUbGBcqGHRaWkcqaIXaqmaaGccqGGVaWldaWadiqaamaabmGabaGaemOBa4Maey4kaSIaeGymaedacaGLOaGaayzkaaGaeiyiaeIaemOBa4MaeiyiaecacaGLBbGaayzxaaaaaa@5465@

We simplify this expression using the identity

(*i*^3/2^*αR*)^2^*J*_0_(*i*^3/2^*αR*) - 2(*i*^3/2^*αR*)*J*_1_(*i*^3/2^*αR*) = -(*i*^3/2^*αR*)^2^*J*_2_(*i*^3/2^*αR*), where *J*_2_(*i*^3/2^*αR*) is the Bessel function of order 2,

∑n=0n=∞(−1)n(i3/2αR/2)2n+2/[(n+2)!n!].
 MathType@MTEF@5@5@+=feaafiart1ev1aaatCvAUfKttLearuWrP9MDH5MBPbIqV92AaeXatLxBI9gBaebbnrfifHhDYfgasaacH8akY=wiFfYdH8Gipec8Eeeu0xXdbba9frFj0=OqFfea0dXdd9vqai=hGuQ8kuc9pgc9s8qqaq=dirpe0xb9q8qiLsFr0=vr0=vr0dc8meaabaqaciaacaGaaeqabaqabeGadaaakeaadaaeWaqaamaabmGabaGaeyOeI0IaeGymaedacaGLOaGaayzkaaWaaWbaaSqabeaacqWGUbGBaaGcdaqadiqaaiabdMgaPnaaCaaaleqabaGaeG4mamJaei4la8IaeGOmaidaaGGacOGae8xSdeMaemOuaiLaei4la8IaeGOmaidacaGLOaGaayzkaaWaaWbaaSqabeaacqaIYaGmcqWGUbGBcqGHRaWkcqaIYaGmaaaabaGaemOBa4Maeyypa0JaeGimaadabaGaemOBa4Maeyypa0JaeyOhIukaniabggHiLdGccqGGVaWldaWadiqaamaabmGabaGaemOBa4Maey4kaSIaeGOmaidacaGLOaGaayzkaaGaeiyiaeIaemOBa4MaeiyiaecacaGLBbGaayzxaaGaeiOla4caaa@554D@

Division of both sides by (*i*^3/2^*αR*)^2 ^gives

*J*_0_(*i*^3/2^*αR*) - 2*J*_1_(*i*^3/2^*αR*)/(*i*^3/2^*αR*) = -*J*_2_(*i*^3/2^*αR*), and substitution gives

Q⌣
 MathType@MTEF@5@5@+=feaafiart1ev1aaatCvAUfKttLearuWrP9MDH5MBPbIqV92AaeXatLxBI9gBaebbnrfifHhDYfgasaacH8akY=wiFfYdH8Gipec8Eeeu0xXdbba9frFj0=OqFfea0dXdd9vqai=hGuQ8kuc9pgc9s8qqaq=dirpe0xb9q8qiLsFr0=vr0=vr0dc8meaabaqaciaacaGaaeqabaqabeGadaaakeaacuWGrbqugaafaaaa@2DF2@ = [*πĂR*^2^*e*^*iωt*^/(*iωρ*)][-*J*_2_(*i*^3/2^*αR*)/*J*_0_(*i*^3/2^*αR*)].     (11)

We define *P*_*Q*_(*i*^3/2^*αR*) as *J*_2_(*i*^3/2^*αR*)/[(*i*^3/2^*αR*)^2^/8] and note that as *R *goes to zero the imaginary part of *P*_*Q*_(*i*^3/2^*αR*) vanishes and |*P*_*Q*_(*i*^3/2^*αR*)| goes to 1. Substitution now gives Q⌣
 MathType@MTEF@5@5@+=feaafiart1ev1aaatCvAUfKttLearuWrP9MDH5MBPbIqV92AaeXatLxBI9gBaebbnrfifHhDYfgasaacH8akY=wiFfYdH8Gipec8Eeeu0xXdbba9frFj0=OqFfea0dXdd9vqai=hGuQ8kuc9pgc9s8qqaq=dirpe0xb9q8qiLsFr0=vr0=vr0dc8meaabaqaciaacaGaaeqabaqabeGadaaakeaacuWGrbqugaafaaaa@2DF2@ = [*πĂR*^4^/(8*μ*)]*e*^*iωt*^*P*_Q_(*i*^3/2^*αR*)/*J*_0_(*i*^3/2^*αR*). This expression is further simplified to

Q⌣=[πA⌣R4/(8μ)]eiωt+iθPQ−iθJ0|PQ(i3/2αR)|/|J0(i3/2αR)|,     (12)
 MathType@MTEF@5@5@+=feaafiart1ev1aaatCvAUfKttLearuWrP9MDH5MBPbIqV92AaeXatLxBI9gBaebbnrfifHhDYfgasaacH8akY=wiFfYdH8Gipec8Eeeu0xXdbba9frFj0=OqFfea0dXdd9vqai=hGuQ8kuc9pgc9s8qqaq=dirpe0xb9q8qiLsFr0=vr0=vr0dc8meaabaqaciaacaGaaeqabaqabeGadaaakeaacuWGrbqugaafaiabg2da9maadmGabaacciGae8hWdaNafmyqaeKbaqbacqWGsbGudaahaaWcbeqaaiabisda0aaakiabc+caVmaabmGabaGaeGioaGJae8hVd0gacaGLOaGaayzkaaaacaGLBbGaayzxaaGaemyzau2aaWbaaSqabeaacqWGPbqAcqWFjpWDcqWG0baDcqGHRaWkcqWGPbqAcqWF4oqCdaWgaaadbaGaemiuaaLaemyuaefabeaaliabgkHiTiabdMgaPjab=H7aXnaaBaaameaacqWGkbGscqaIWaamaeqaaaaakmaaemGabaGaemiuaa1aaSbaaSqaaiabdgfarbqabaGcdaqadiqaaiabdMgaPnaaCaaaleqabaGaeG4mamJaei4la8IaeGOmaidaaOGae8xSdeMaemOuaifacaGLOaGaayzkaaaacaGLhWUaayjcSdGaei4la8YaaqWaceaacqWGkbGsdaWgaaWcbaGaeGimaadabeaakmaabmGabaGaemyAaK2aaWbaaSqabeaacqaIZaWmcqGGVaWlcqaIYaGmaaGccqWFXoqycqWGsbGuaiaawIcacaGLPaaaaiaawEa7caGLiWoacqGGSaalcaWLjaGaaCzcamaabmGabaGaeGymaeJaeGOmaidacaGLOaGaayzkaaaaaa@7103@

where *θ*_*PQ *_is the argument of *P*_*Q*_(*i*^3/2^*αR*) and *θ*_*j*0 _is the argument of *J*_0_(*i*^3/2^*αR*).

The shear force (per unit area) at the inner wall of the tube is -*μ*[*∂u*/*∂r*|_*r*=*R *_= -[*Ăμ*/(*ρiω*)]e^*iωt*^*i*^3/2^*αJ*_-1_(*i*^3/2^*αR*)/*J*_0_(*i*^3/2^*αR*), where *i*^3/2^*αJ*_-1_(*i*^3/2^*αR*) = *dJ*_0_(*i*^3/2^*αr*)/*dr*|_*r*=*R*_. We note that -*J*_-1_(*i*^3/2^*αR*) = *J*_1_(*i*^3/2^*αR*), which is written as (*i*^3/2^*αR*/2)*P*_*S*_(*i*^3/2^*αR*). Substitution now gives the expression for the shear force (per unit area), [*Ăe*^*iωt*^*R*/2]*P*_*S*_(*i*^3/2^*αR*)/*J*_0_(*i*^3/2^*αR*). This expression is further simplified to

−μ[∂u/∂r]r=R=[A⌣R/2]eiωt+iθPS−iθJ0|PS(i3/2αR)|/|J0(i3/2αR)|,     (13)
 MathType@MTEF@5@5@+=feaafiart1ev1aaatCvAUfKttLearuWrP9MDH5MBPbIqV92AaeXatLxBI9gBaebbnrfifHhDYfgasaacH8akY=wiFfYdH8Gipec8Eeeu0xXdbba9frFj0=OqFfea0dXdd9vqai=hGuQ8kuc9pgc9s8qqaq=dirpe0xb9q8qiLsFr0=vr0=vr0dc8meaabaqaciaacaGaaeqabaqabeGadaaakeaacqGHsisliiGacqWF8oqBdaWadiqaaiabgkGi2kabdwha1jabc+caViabgkGi2kabdkhaYbGaay5waiaaw2faamaaBaaaleaacqWGYbGCcqGH9aqpcqWGsbGuaeqaaOGaeyypa0ZaamWaceaacuWGbbqqgaafaiabdkfasjabc+caViabikdaYaGaay5waiaaw2faaiabdwgaLnaaCaaaleqabaGaemyAaKMae8xYdCNaemiDaqNaey4kaSIaemyAaKMae8hUde3aaSbaaWqaaiabdcfaqjabdofatbqabaWccqGHsislcqWGPbqAcqWF4oqCdaWgaaadbaGaemOsaOKaeGimaadabeaaaaGcdaabdiqaaiabdcfaqnaaBaaaleaacqWGtbWuaeqaaOWaaeWaceaacqWGPbqAdaahaaWcbeqaaiabiodaZiabc+caViabikdaYaaakiab=f7aHjabdkfasbGaayjkaiaawMcaaaGaay5bSlaawIa7aiabc+caVmaaemGabaGaemOsaO0aaSbaaSqaaiabicdaWaqabaGcdaqadiqaaiabdMgaPnaaCaaaleqabaGaeG4mamJaei4la8IaeGOmaidaaOGae8xSdeMaemOuaifacaGLOaGaayzkaaaacaGLhWUaayjcSdGaeiilaWIaaCzcaiaaxMaadaqadiqaaiabigdaXiabiodaZaGaayjkaiaawMcaaaaa@7894@

where *θ*_*PS *_is the argument of *P*_*S*_(*i*^3/2^*αR*). Note that *P*_*S*_(*i*^3/2^*αR*) is a function with an imaginary part that vanishes and a modulus that approaches 1 as *R *approaches 0.

The final step in the description of the arterial pressure gradient is to express it as a sum of a forward-pumping gradient and a purely oscillatory gradient. In large human arteries under normal physiological conditions, pressure cycles from approximately 120 mm Hg (systolic) to approximately 80 mm Hg (diastolic). Pressure in the terminal arterioles is much lower than 80 mm Hg. These pressures suggest a model where there is a forward-pumping pressure gradient, *Ã*, plus an oscillating pressure gradient, *Ăe*^*iωt*^. The flow velocity of this forward-pumping, pulsatile model is the sum of the solutions for the constant gradient model and the oscillating gradient model. Similarly, the flow rate in the tube and the shear force at the inner wall of the tube are the sums of the flow rates and the shear forces, respectively, of the constant gradient model and the oscillating gradient model.

### Flow in an elastic tube

Now consider flow in an elastic tube where the radius is constant but the inside surface moves in response to the pull of adjacent fluid. The thickness of the wall is denoted *h*, and wall tissue density is denoted *ρ*_*w*_. The displacement of a point on the inside wall of the tube from the locus when the oscillatory component of force is identically 0 is denoted *Z*, and the coefficient of deformation relating *Z *to the force per unit area along the inside wall is *K*.

The model considered in this section differs from the Womersley model in how the elastic tube responds to shear force on the inner wall. In the Womersley model, the outer wall is not connected to surrounding structures. The full thickness of the wall moves in response to shear force, and regions of relatively high force stretch upstream regions of the wall and compress downstream regions. In the model analyzed below, the outer wall is tethered by branching arteries, and its movement is further restricted by contact with adjacent tissues. The tube matrix between the inner and outer wall is modeled as elastic tissue.

We first consider the pressure gradient *Ăe*^*iωt*^. From Equation (9), the expression for *ŭ *is again [*Ă*/(*ρiω*)]*e*^*iωt *^+ *B*_1,0_*e*^*iωt*^*J*_0_(*i*^3/2^*αr*), where *B*_1,0 _is determined by the boundary condition requiring the velocity of fluid at the wall surface to equal the velocity of the wall surface. The solution for wall displacement, *Z*, is periodic with period 2*π*/*ω*. Therefore, the position of the inner wall of the tube is described by a Fourier series

Z=∑n=−∞n=+∞Cneinωt.
 MathType@MTEF@5@5@+=feaafiart1ev1aaatCvAUfKttLearuWrP9MDH5MBPbIqV92AaeXatLxBI9gBaebbnrfifHhDYfgasaacH8akY=wiFfYdH8Gipec8Eeeu0xXdbba9frFj0=OqFfea0dXdd9vqai=hGuQ8kuc9pgc9s8qqaq=dirpe0xb9q8qiLsFr0=vr0=vr0dc8meaabaqaciaacaGaaeqabaqabeGadaaakeaacqWGAbGwcqGH9aqpdaaeWaqaaiabdoeadnaaBaaaleaacqWGUbGBaeqaaOGaemyzau2aaWbaaSqabeaacqWGPbqAcqWGUbGBiiGacqWFjpWDcqWG0baDaaGccqGGUaGlaSqaaiabd6gaUjabg2da9iabgkHiTiabg6HiLcqaaiabd6gaUjabg2da9iabgUcaRiabg6HiLcqdcqGHris5aaaa@4595@

The condition requiring the velocity of the wall to equal the velocity of the adjacent fluid gives

∑n=−∞n=+∞Cninωeinωt=[A⌣/(ρiω)]eiωt+B1,0eiωtJ0(i3/2αR).
 MathType@MTEF@5@5@+=feaafiart1ev1aaatCvAUfKttLearuWrP9MDH5MBPbIqV92AaeXatLxBI9gBaebbnrfifHhDYfgasaacH8akY=wiFfYdH8Gipec8Eeeu0xXdbba9frFj0=OqFfea0dXdd9vqai=hGuQ8kuc9pgc9s8qqaq=dirpe0xb9q8qiLsFr0=vr0=vr0dc8meaabaqaciaacaGaaeqabaqabeGadaaakeaadaaeWaqaaiabdoeadnaaBaaaleaacqWGUbGBaeqaaOGaemyAaKMaemOBa4gcciGae8xYdCNaemyzau2aaWbaaSqabeaacqWGPbqAcqWGUbGBcqWFjpWDcqWG0baDaaGccqGH9aqpdaWadiqaaiqbdgeabzaauaGaei4la8YaaeWaceaacqWFbpGCcqWGPbqAcqWFjpWDaiaawIcacaGLPaaaaiaawUfacaGLDbaacqWGLbqzdaahaaWcbeqaaiabdMgaPjab=L8a3jabdsha0baakiabgUcaRiabdkeacnaaBaaaleaacqaIXaqmcqGGSaalcqaIWaamaeqaaOGaemyzau2aaWbaaSqabeaacqWGPbqAcqWFjpWDcqWG0baDaaGccqWGkbGsdaWgaaWcbaGaeGimaadabeaakmaabmGabaGaemyAaK2aaWbaaSqabeaacqaIZaWmcqGGVaWlcqaIYaGmaaGccqWFXoqycqWGsbGuaiaawIcacaGLPaaacqGGUaGlaSqaaiabd6gaUjabg2da9iabgkHiTiabg6HiLcqaaiabd6gaUjabg2da9iabgUcaRiabg6HiLcqdcqGHris5aaaa@6F59@

Equating the coefficients of *e*^*inωt *^leads to *C*_1_= (*Ă*/*ρ*)/(*iω*)^2 ^+ *B*_1,0_*J*_0_(*i*^3/2^*αR*)/(*iω*), and from the definition of *Z*, it follows that *C*_0 _= 0. Furthermore, for *n *> 1 and for *n *< 0 *C*_*n *_= 0. Consequently, we have

*Z *= {(*Ă*/*ρ*)/(*iω*)^2 ^+ *B*_1,0_*J*_0_(*i*^3/2^*αR*)/(*iω*)}*e*^*inωt*^.     (14)

If the outer wall is assumed to be stationary, the average velocity of the wall is described by *iω C*_1_*e*^*iωt*^/2, and the requirement that the force (per unit surface area) on a point on the wall, -*μ*[*∂v*/*∂r*|_*r*=*R*_-*KC*_1_*e*^*iωt*^, equals the rate of change of wall momentum (per unit wall surface area), (*hρ*_*w*_/2)(*iω*)^2^*C*_1_*e*^*iωt*^, gives

-*μ*[*∂B*_1,0_*e*^*iωt*^*J*_0_(*i*^3/2^*αr*)/*∂r*|_*r*=*R*_] - *KC*_1_*e*^*iωt *^= (*hρ*_*w*_/2)(*iω*)^2^*C*_1_*e*^*iωt*^.

Substitution for *C*_1 _gives

*μB*_1,0_*e*^*iωt*^*i*^3/2^*αJ*_-1_(*i*^3/2^*αR*)*e*^*iωt*^/[-*K*/(*iω*) - (*hρ*_*w*_/2)(*iω*)]

= [*Ă*/(*iω*)]*e*^*iωt *^+ *B*_1,0_*e*^*iωt*^*J*_0_(*i*^3/2^*αR*),

where *i*^3/2^*αJ*_-1_(*i*^3/2^*αR*) = *∂J*_0_(*i*^3/2^*αr*)/*∂r*|_*r*=*R*_. Consequently,

*B*_1,0 _= - [*Ă*/(*ρiω*)]/{*J*_0_(*i*^3/2^*αR*) - *μ*[*i*^3/2^*αJ*_-1_(*i*^3/2^*αR*)]/(-*K*/(*iω*) - (*iω*)*hρ*_*w*_/2)},     (15)

and

*ŭ *= [*Ă*/(*ρiω*)]*e*^*iωt*^{1 - *J*_0_(*i*^3/2^*αr*)/[*J*_0_(*i*^3/2^*αR*)

+*μi*^3/2^*αJ*_1_(*i*^3/2^*αR*)/(*K*/(*iω*) + (*iω*)*hρ*_*w*_/2)]}.     (16)

Now, consider the relationship between the thickness *h *and the elastic coefficient *K *of the wall. Using the analogy of a sheet of rubber with thickness *h*, we can write *K *= κ/*h*, where κ is a constant. Substituting κ/*h *for *K *and -*J*_1_(*i*^3/2^*αR*) for *J*_-1_(*i*^3/2^*αR*) in Equation (15) gives

*ŭ *= [*Ă*/(*ρiω*)]*e*^*iωt*^{1 - *J*_0_(*i*^3/2^*αr*)/[*J*_0_(*i*^3/2^*αR*) + *DP*_*S*_(*i*^3/2^*αR*)]},     (17)

where

*D *= (*μ*/*κ*)(*h*/*R*)(*iω*)(*i*^3/2^*αR*)^2^/2/(1 + (*h*/*κ*)(*iω*)^2^*hρ*_*w*_/2).     (18)

Integration over the cross-sectional area of the tube now gives

Q⌣
 MathType@MTEF@5@5@+=feaafiart1ev1aaatCvAUfKttLearuWrP9MDH5MBPbIqV92AaeXatLxBI9gBaebbnrfifHhDYfgasaacH8akY=wiFfYdH8Gipec8Eeeu0xXdbba9frFj0=OqFfea0dXdd9vqai=hGuQ8kuc9pgc9s8qqaq=dirpe0xb9q8qiLsFr0=vr0=vr0dc8meaabaqaciaacaGaaeqabaqabeGadaaakeaacuWGrbqugaafaaaa@2DF2@= [*πĂR*^2^/(*ρiω*)]*e*^*iωt*^{1 - *P*_*S*_(*i*^3/2^*αR*)/[*J*_0_(*i*^3/2^*αR*) + *DP*_*S*_(*i*^3/2^*αR*)]}.     (19)

From Equation (17), the shear force (per unit area) at the wall of the elastic tube is

-*μ*[*∂u*/*∂r*|_*r*=*R *_= [*Ăμ*/(*ρiω*)]*e*^*iωt*^(-*i*^3/2^*α*)(*i*^3/2^*αR*/2)*P*_*S*_(*i*^3/2^*αR*)/[*J*_0_(*i*^3/2^*αR*) + *DP*_*S*_(*i*^3/2^*αR*)].

This expression is further simplified to

-μ[*∂u*/*∂r*|_*r*=*R *_= [*ĂR*/2]*e*^*iωt*^*P*_*S*_(*i*^3/2^*αR*)/[*J*_0_(*i*^3/2^*αR*) + *DP*_*S*_(*i*^3/2^*αR*)].     (20)

We note that, as *D *goes to 0, the above expressions for flow velocity, flow rate and shear force in the elastic tube model approach the values given by Equation (10), Equation (11) and Equation (13), respectively, derived for the rigid tube model. A bound on *D *can be derived from the expression for *Z*, Equation (14), which is simplified by substituting the value of *B*_1,0 _from Equation (15) and the definition of *D *from Equation (18) to give

*Z *= [*Ă*/(*ρω*^2^)]*e*^*iωt*^*DP*_*S*_(*i*^3/2^*αR*)/[*J*_0_(*i*^3/2^*αR*) + *DP*_*S*_(*i*^3/2^*αR*)].

We now divide Equation (20) by -*iωπR*^2^*Z *to give

*D *= {[*J*_0_(*i*^3/2^*αR*) - *P*_*S*_(*i*^3/2^*αR*)]/*P*_*S*_(*i*^3/2^*αR*)}/[Q⌣
 MathType@MTEF@5@5@+=feaafiart1ev1aaatCvAUfKttLearuWrP9MDH5MBPbIqV92AaeXatLxBI9gBaebbnrfifHhDYfgasaacH8akY=wiFfYdH8Gipec8Eeeu0xXdbba9frFj0=OqFfea0dXdd9vqai=hGuQ8kuc9pgc9s8qqaq=dirpe0xb9q8qiLsFr0=vr0=vr0dc8meaabaqaciaacaGaaeqabaqabeGadaaakeaacuWGrbqugaafaaaa@2DF2@/(*iωπR*^2^*Z*) - 1],

which implies

*D *≤ [|*J*_0_(*i*^3/2^*αR*)|/|*P*_*S*_(*i*^3/2^*αR*)|+1]/|Q⌣
 MathType@MTEF@5@5@+=feaafiart1ev1aaatCvAUfKttLearuWrP9MDH5MBPbIqV92AaeXatLxBI9gBaebbnrfifHhDYfgasaacH8akY=wiFfYdH8Gipec8Eeeu0xXdbba9frFj0=OqFfea0dXdd9vqai=hGuQ8kuc9pgc9s8qqaq=dirpe0xb9q8qiLsFr0=vr0=vr0dc8meaabaqaciaacaGaaeqabaqabeGadaaakeaacuWGrbqugaafaaaa@2DF2@/(*iωπR*^2^*Z*) - 1|.

We can set an upper bound on *D *by setting an upper bound on *Z*. For example, a reasonable assumption is that the maximum value of *Z *is bounded by *h*/2. This condition limits the movement of the inner surface of the tube during a cycle of pulsatile flow to a distance no greater than the thickness of the vessel wall. In small muscular arteries and arterioles, *h *is approximately equal to or slightly less than *R *[8]. As *R *increases, *h/R *decreases to a value of approximately 0.2 for the aorta [9]. The viscosity of blood at a shear gradient of 100/s is approximately 0.033 dyne-s/cm^2 ^[10], and the density is approximately 1.06 g/cm^3^. Therefore, at a heart rate of 1/s, *α*^2 ^is approximately 32/cm^2^.

The rate of arterial blood flow in the proximal aorta is equal to the cardiac output, which in humans is approximately 70 ml/s. The velocity ranges from approximately 100 cm/s in early systole to 0 or a slightly negative value in diastole. In our oscillatory, forward-pumping model, total blood flow in the human aorta is described by *Q *= Q˜
 MathType@MTEF@5@5@+=feaafiart1ev1aaatCvAUfKttLearuWrP9MDH5MBPbIqV92AaeXatLxBI9gBaebbnrfifHhDYfgasaacH8akY=wiFfYdH8Gipec8Eeeu0xXdbba9frFj0=OqFfea0dXdd9vqai=hGuQ8kuc9pgc9s8qqaq=dirpe0xb9q8qiLsFr0=vr0=vr0dc8meaabaqaciaacaGaaeqabaqabeGadaaakeaacuWGrbqugaacaaaa@2DE6@ + Q⌣
 MathType@MTEF@5@5@+=feaafiart1ev1aaatCvAUfKttLearuWrP9MDH5MBPbIqV92AaeXatLxBI9gBaebbnrfifHhDYfgasaacH8akY=wiFfYdH8Gipec8Eeeu0xXdbba9frFj0=OqFfea0dXdd9vqai=hGuQ8kuc9pgc9s8qqaq=dirpe0xb9q8qiLsFr0=vr0=vr0dc8meaabaqaciaacaGaaeqabaqabeGadaaakeaacuWGrbqugaafaaaa@2DF2@, where Q˜
 MathType@MTEF@5@5@+=feaafiart1ev1aaatCvAUfKttLearuWrP9MDH5MBPbIqV92AaeXatLxBI9gBaebbnrfifHhDYfgasaacH8akY=wiFfYdH8Gipec8Eeeu0xXdbba9frFj0=OqFfea0dXdd9vqai=hGuQ8kuc9pgc9s8qqaq=dirpe0xb9q8qiLsFr0=vr0=vr0dc8meaabaqaciaacaGaaeqabaqabeGadaaakeaacuWGrbqugaacaaaa@2DE6@ = 70 ml/s and Q⌣
 MathType@MTEF@5@5@+=feaafiart1ev1aaatCvAUfKttLearuWrP9MDH5MBPbIqV92AaeXatLxBI9gBaebbnrfifHhDYfgasaacH8akY=wiFfYdH8Gipec8Eeeu0xXdbba9frFj0=OqFfea0dXdd9vqai=hGuQ8kuc9pgc9s8qqaq=dirpe0xb9q8qiLsFr0=vr0=vr0dc8meaabaqaciaacaGaaeqabaqabeGadaaakeaacuWGrbqugaafaaaa@2DF2@ = (70 ml/s)*e*^*iωt*^. From the value of *R*, approximately 1 cm, *αR *is approximately 5, and h is approximately 0.2 cm. Therefore, from Table 1, |*J*_0_(*i*^3/2^*αR*)|/|*P*_*S*_(*i*^3/2^*αR*)| is approximately equal to (but less than) 3. From the bounding of *Z *assumption, the quantity |Q⌣
 MathType@MTEF@5@5@+=feaafiart1ev1aaatCvAUfKttLearuWrP9MDH5MBPbIqV92AaeXatLxBI9gBaebbnrfifHhDYfgasaacH8akY=wiFfYdH8Gipec8Eeeu0xXdbba9frFj0=OqFfea0dXdd9vqai=hGuQ8kuc9pgc9s8qqaq=dirpe0xb9q8qiLsFr0=vr0=vr0dc8meaabaqaciaacaGaaeqabaqabeGadaaakeaacuWGrbqugaafaaaa@2DF2@/(*iωπR*^2^*Z*)| is greater than |2Q⌣
 MathType@MTEF@5@5@+=feaafiart1ev1aaatCvAUfKttLearuWrP9MDH5MBPbIqV92AaeXatLxBI9gBaebbnrfifHhDYfgasaacH8akY=wiFfYdH8Gipec8Eeeu0xXdbba9frFj0=OqFfea0dXdd9vqai=hGuQ8kuc9pgc9s8qqaq=dirpe0xb9q8qiLsFr0=vr0=vr0dc8meaabaqaciaacaGaaeqabaqabeGadaaakeaacuWGrbqugaafaaaa@2DF2@/(*iωπR*^2^*h)*|, which is in turn greater than 201. Therefore, 1/|Q⌣
 MathType@MTEF@5@5@+=feaafiart1ev1aaatCvAUfKttLearuWrP9MDH5MBPbIqV92AaeXatLxBI9gBaebbnrfifHhDYfgasaacH8akY=wiFfYdH8Gipec8Eeeu0xXdbba9frFj0=OqFfea0dXdd9vqai=hGuQ8kuc9pgc9s8qqaq=dirpe0xb9q8qiLsFr0=vr0=vr0dc8meaabaqaciaacaGaaeqabaqabeGadaaakeaacuWGrbqugaafaaaa@2DF2@/(*iωπR*^2^*Z*) - 1| is less than 1/200, and *D *is less than 2 × 10^-2^.

**Table 1:** Values of |*J*_0_(*i*^3/2^*αR*)|, |*P*_*S*_(*i*^3/2^*αR*)|/|*J*_0_(*i*^3/2^*αR*)|, |*P*_*Q*_*(i*^3/2^*αR*)|/|*J*_0_(*i*^3/2^*αR*)| and *θ*_*d *_= -*θ*_*PQ *_+ *θ*_*J*0 _(in degrees).

*αR*	|*J*_0_(*i*^3/2^*αR*)|	|*P*_*S*_(*i*^3/2^*αR*)|	|PS(i3/2αR)||J0(i3/2αR)| MathType@MTEF@5@5@+=feaafiart1ev1aaatCvAUfKttLearuWrP9MDH5MBPbIqV92AaeXatLxBI9gBaebbnrfifHhDYfgasaacH8akY=wiFfYdH8Gipec8Eeeu0xXdbba9frFj0=OqFfea0dXdd9vqai=hGuQ8kuc9pgc9s8qqaq=dirpe0xb9q8qiLsFr0=vr0=vr0dc8meaabaqaciaacaGaaeqabaqabeGadaaakeaadaWcaaqaamaaemGabaGaemiuaa1aaSbaaSqaaiabdofatbqabaGcdaqadiqaaiabdMgaPnaaCaaaleqabaGaeG4mamJaei4la8IaeGOmaidaaGGacOGae8xSdeMaemOuaifacaGLOaGaayzkaaaacaGLhWUaayjcSdaabaWaaqWaceaacqWGkbGsdaWgaaWcbaGaeGimaadabeaakmaabmGabaGaemyAaK2aaWbaaSqabeaacqaIZaWmcqGGVaWlcqaIYaGmaaGccqWFXoqycqWGsbGuaiaawIcacaGLPaaaaiaawEa7caGLiWoaaaaaaa@493F@	|PQ(i3/2αR)||J0(i3/2αR)| MathType@MTEF@5@5@+=feaafiart1ev1aaatCvAUfKttLearuWrP9MDH5MBPbIqV92AaeXatLxBI9gBaebbnrfifHhDYfgasaacH8akY=wiFfYdH8Gipec8Eeeu0xXdbba9frFj0=OqFfea0dXdd9vqai=hGuQ8kuc9pgc9s8qqaq=dirpe0xb9q8qiLsFr0=vr0=vr0dc8meaabaqaciaacaGaaeqabaqabeGadaaakeaadaWcaaqaamaaemGabaGaemiuaa1aaSbaaSqaaiabdgfarbqabaGcdaqadiqaaiabdMgaPnaaCaaaleqabaGaeG4mamJaei4la8IaeGOmaidaaGGacOGae8xSdeMaemOuaifacaGLOaGaayzkaaaacaGLhWUaayjcSdaabaWaaqWaceaacqWGkbGsdaWgaaWcbaGaeGimaadabeaakmaabmGabaGaemyAaK2aaWbaaSqabeaacqaIZaWmcqGGVaWlcqaIYaGmaaGccqWFXoqycqWGsbGuaiaawIcacaGLPaaaaiaawEa7caGLiWoaaaaaaa@493B@	-*θ*_*PQ *_+ *θ*_*J*0_
0.00	1.000000	1.000000	1.000000	1.000000	0.000000
0.25	1.000061	1.00001	0.999949	0.999942	0.596807
0.50	1.000976	1.000163	0.999187	0.999079	2.385789
0.75	1.004934	1.000824	0.99591	0.995364	5.354071
1.00	1.015525	1.002602	0.987275	0.985567	9.452664
1.25	1.037563	1.006346	0.969913	0.965839	14.56122
1.50	1.076683	1.013132	0.940975	0.932856	20.45769
1.75	1.138718	1.02425	0.899476	0.885319	26.81781
2.00	1.229006	1.041167	0.847162	0.824936	33.26157
2.25	1.351958	1.065491	0.78811	0.756052	39.43308
2.50	1.511077	1.098913	0.727238	0.684073	45.0714
2.75	1.709413	1.143157	0.668742	0.613766	50.03823
3.00	1.950193	1.199938	0.615292	0.548354	54.30262
3.25	2.237433	1.270948	0.568039	0.48947	57.90541
3.50	2.576414	1.357868	0.527038	0.437549	60.9244
3.75	2.97404	1.462421	0.491729	0.392306	63.44954
4.00	3.439118	1.586448	0.461295	0.353099	65.56827
4.25	3.982607	1.732001	0.434891	0.319165	67.3584
4.50	4.617878	1.901445	0.411757	0.289747	68.88554
4.75	5.361012	2.097556	0.391261	0.264156	70.20298
5.00	6.231163	2.323623	0.372904	0.241795	71.35285

We can also use data on blood flow velocity to calculate a bound on *D *using the equation Q⌣
 MathType@MTEF@5@5@+=feaafiart1ev1aaatCvAUfKttLearuWrP9MDH5MBPbIqV92AaeXatLxBI9gBaebbnrfifHhDYfgasaacH8akY=wiFfYdH8Gipec8Eeeu0xXdbba9frFj0=OqFfea0dXdd9vqai=hGuQ8kuc9pgc9s8qqaq=dirpe0xb9q8qiLsFr0=vr0=vr0dc8meaabaqaciaacaGaaeqabaqabeGadaaakeaacuWGrbqugaafaaaa@2DF2@ = *πR*^2^*Ave*{*ŭ*} where *Ave*{*ŭ*} denotes the cross-sectional average value of *ŭ*. For the lumbar artery, a medium-sized artery with *R *approximately equal to 0.1 cm, *αR *is approximately 0.5. (An artery of medium size is defined here as one with a radius between 0.15 cm and 0.015 cm.) From Table 1, |*P*_*S*_(*i*^3/2^*αR*)| is very close to 1. From the first term of the Taylor's series for *J*_0_(*i*^3/2^*αR*) - *P*_*S*_(*i*^3/2^*αR*), |*J*_0_(*i*^3/2^*αR*) - *P*_*S*_(*i*^3/2^*αR*)| is approximately 2^-5^. For the lumbar artery, *Ave*{*ŭ*} is approximately 10 cm/s [11]. Therefore, in this example *D *is less than 10^-3^.

Finally, we note from Equation (18) that *D *is a decreasing function of both *h *and *R*. Because both *h *and *R *decrease from the aorta to the terminal arterioles, *D *is clearly very small throughout the arterial system. Consequently, the expressions for the rate of blood flow and the shear force on the inner surface of the wall of an elastic tube are closely approximated by the corresponding expressions, Equation (11) and Equation (13), for the rigid tube.

### Shear force and Murray's Law

Consider a vessel of radius *R*_*k *_that branches into *η *vessels of radius *R*_*k*+1_. Clearly, the average rate of flow in the parent artery is *η *times the average rate of flow in each daughter artery. Similarly, the peak rate of flow in the parent artery is *η *times the peak rate of flow in each daughter artery. From Equation (3) and Equation (12), this condition is expressed as

(πRk4/8μ)[A˜k+A⌣k|PQ(i3/2αRk)|/|J0(i3/2αRk)|]=η(πRk+14/8μ)[A˜k+1+A⌣k+1|PQ(i3/2αRk+1)|/|J0(i3/2αRk+1)|].     (21)
 MathType@MTEF@5@5@+=feaafiart1ev1aaatCvAUfKttLearuWrP9MDH5MBPbIqV92AaeXatLxBI9gBaebbnrfifHhDYfgasaacH8akY=wiFfYdH8Gipec8Eeeu0xXdbba9frFj0=OqFfea0dXdd9vqai=hGuQ8kuc9pgc9s8qqaq=dirpe0xb9q8qiLsFr0=vr0=vr0dc8meaabaqaciaacaGaaeqabaqabeGadaaakeaafaqaaeGabaaabaWaaeWaceaaiiGacqWFapaCcqWGsbGudaqhaaWcbaGaem4AaSgabaGaeGinaqdaaOGaei4la8IaeGioaGJae8hVd0gacaGLOaGaayzkaaGaei4waSLafmyqaeKbaGaadaWgaaWcbaGaem4AaSgabeaakiabgUcaRiqbdgeabzaauaWaaSbaaSqaaiabdUgaRbqabaGcdaabdiqaaiabdcfaqnaaBaaaleaacqWGrbquaeqaaOWaaeWaceaacqWGPbqAdaahaaWcbeqaaiabiodaZiabc+caViabikdaYaaakiab=f7aHjabdkfasnaaBaaaleaacqWGRbWAaeqaaaGccaGLOaGaayzkaaaacaGLhWUaayjcSdGaei4la8YaaqWaceaacqWGkbGsdaWgaaWcbaGaeGimaadabeaakmaabmGabaGaemyAaK2aaWbaaSqabeaacqaIZaWmcqGGVaWlcqaIYaGmaaGccqWFXoqycqWGsbGudaWgaaWcbaGaem4AaSgabeaaaOGaayjkaiaawMcaaaGaay5bSlaawIa7aiabc2faDbqaaiabg2da9iab=D7aOnaabmGabaGae8hWdaNaemOuai1aa0baaSqaaiabdUgaRjabgUcaRiabigdaXaqaaiabisda0aaakiabc+caViabiIda4iab=X7aTbGaayjkaiaawMcaaiabcUfaBjqbdgeabzaaiaWaaSbaaSqaaiabdUgaRjabgUcaRiabigdaXaqabaGccqGHRaWkcuWGbbqqgaafamaaBaaaleaacqWGRbWAcqGHRaWkcqaIXaqmaeqaaOWaaqWaceaacqWGqbaudaWgaaWcbaGaemyuaefabeaakmaabmGabaGaemyAaK2aaWbaaSqabeaacqaIZaWmcqGGVaWlcqaIYaGmaaGccqWFXoqycqWGsbGudaWgaaWcbaGaem4AaSMaey4kaSIaeGymaedabeaaaOGaayjkaiaawMcaaaGaay5bSlaawIa7aiabc+caVmaaemGabaGaemOsaO0aaSbaaSqaaiabicdaWaqabaGcdaqadiqaaiabdMgaPnaaCaaaleqabaGaeG4mamJaei4la8IaeGOmaidaaOGae8xSdeMaemOuai1aaSbaaSqaaiabdUgaRjabgUcaRiabigdaXaqabaaakiaawIcacaGLPaaaaiaawEa7caGLiWoacqGGDbqxcqGGUaGlaaGaaCzcaiaaxMaadaqadiqaaiabikdaYiabigdaXaGaayjkaiaawMcaaaaa@A5E0@

From Equation (4) and Equation (13), the SFR hypothesis states that

(*R*_*k*_/2)[*Ã*_*k *_+ *Ă*_*k*_|*P*_*S*_(*i*^3/2^*αR*_*k*_)|/|*J*_0_(*i*^3/2^*αR*_*k*_)|]

= (*R*_*k*+1_/2)[*Ã*_*k*+1 _+ *Ă*_*k*+1_|*P*_*S*_(*i*^3/2^*αR*_*k*+1_)|/|*J*_0_(*i*^3/2^*αR*_*k*+1_)|].     (22)

The approximations |*P*_*Q*_(*i*^3/2^*αR*)|/|*J*_0_(*i*^3/2^*αR*)| = 1 and |*P*_*S*_(*αR*)|/|*J*_0_(*i*^3/2^*αR*)| = 1 now lead to Murray's Law, Rk3=ηRk+13
 MathType@MTEF@5@5@+=feaafiart1ev1aaatCvAUfKttLearuWrP9MDH5MBPbIqV92AaeXatLxBI9gBaebbnrfifHhDYfgasaacH8akY=wiFfYdH8Gipec8Eeeu0xXdbba9frFj0=OqFfea0dXdd9vqai=hGuQ8kuc9pgc9s8qqaq=dirpe0xb9q8qiLsFr0=vr0=vr0dc8meaabaqaciaacaGaaeqabaqabeGadaaakeaacqWGsbGudaqhaaWcbaGaem4AaSgabaGaeG4mamdaaOGaeyypa0dcciGae83TdGMaemOuai1aa0baaSqaaiabdUgaRjabgUcaRiabigdaXaqaaiabiodaZaaaaaa@389B@. The above argument is easily modified for the general case, i.e., when an artery of radius *R*_*k *_branches into *η *daughter arteries of radius *R*_*k*+1,1_,*R*_*k*+1,2_,...*R*_*k*+1,η_. The general form of Murray's Law is Rk3=Rk+1,13+Rk+1,23+...+Rk+1,η3
 MathType@MTEF@5@5@+=feaafiart1ev1aaatCvAUfKttLearuWrP9MDH5MBPbIqV92AaeXatLxBI9gBaebbnrfifHhDYfgasaacH8akY=wiFfYdH8Gipec8Eeeu0xXdbba9frFj0=OqFfea0dXdd9vqai=hGuQ8kuc9pgc9s8qqaq=dirpe0xb9q8qiLsFr0=vr0=vr0dc8meaabaqaciaacaGaaeqabaqabeGadaaakeaacqWGsbGudaqhaaWcbaGaem4AaSgabaGaeG4mamdaaOGaeyypa0JaemOuai1aa0baaSqaaiabdUgaRjabgUcaRiabigdaXiabcYcaSiabigdaXaqaaiabiodaZaaakiabgUcaRiabdkfasnaaDaaaleaacqWGRbWAcqGHRaWkcqaIXaqmcqGGSaalcqaIYaGmaeaacqaIZaWmaaGccqGHRaWkcWaGejOla4Iamairc6caUiadasKGUaGlcqGHRaWkcqWGsbGudaqhaaWcbaGaem4AaSMaey4kaSIaeGymaeJaeiilaWccciGae83TdGgabaGaeG4mamdaaaaa@4FE5@. When |*P*_*Q*_(*i*^3/2^*αR*)|/|*J*_0_(*i*^3/2^*αR*)| and |*P*_*S*_(*i*^3/2^*αR*)|/|*J*_0_(*i*^3/2^*αR*)| differ significantly from 1, Murray's Law can be derived as an approximation as long as |*P*_*S*_(*i*^3/2^*αR*)|/|*J*_0_(*i*^3/2^*αR*)| is approximately equal to |*P*_*Q*_(*αR*)|/|*J*_0_(*i*^3/2^*αR*)|. Table 1 shows that these ratios are approximately equal for values of *αR *as large as 2.5. Therefore, the SFR hypothesis leads to the conclusion that Murray's Law is a good approximation for all but the largest arteries.

**Figure 1:**
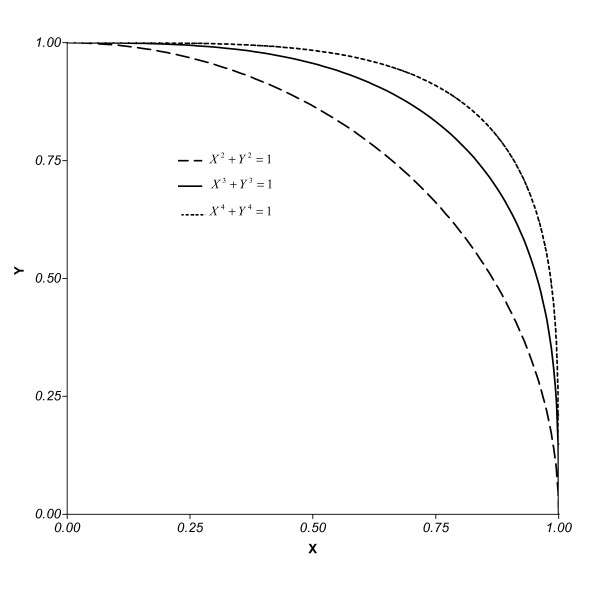
Graphs of three scaling "laws" described by an equation of the form *X*^*M *^+ *Y*^*M *^= 1 where *X *= *R*_*k*+1,1_/*R*_*k*_, *Y *= *R*_*k*+1,2_/*R*_*k *_and *M *> 0.

The statement that Murray's Law is a good approximation has little meaning for highly asymmetric branching. This point can be illustrated by considering a bifurcating artery and noting that Murray's Law for bifurcation can be stated as *X*^3 ^+ *Y*^3 ^= 1, where *X *= *R*_*k*+1,1_/*R*_*k *_and *Y *= *R*_*k*+1,2_/*R*_*k*_. Figure 1 shows that, for *X *≤ 1/2 or *Y *≤ 1/2, values satisfying Murray's Law are approximately equal to values satisfying a second-power law or a fourth-power law. This figure shows that, as branching becomes more and more asymmetric, all laws of the form *X*^*M *^+ *Y*^*M *^= 1, where *M *> 1, give nearly identical predictions for the scaling of vessel radii [12].

### Murray's principle of energy minimization

In a model for the scaling of the mammalian basal metabolic rate (BMR), West et al. used Womersley's model and Murray's principle of energy minimization to argue that the exponent 3 in Murray's Law is replaced by the exponent 2 for large and medium arteries [13]. The claim of second-power scaling is a crucial step in their derivation of a 3/4-power allometric scaling relationship for mammalian BMR. To do this, they argued that flow velocity is independent of vessel radius for medium and large arteries.

It is remarkable that West et al. did not base their energy calculation on the product of pressure gradient and blood flow, *Q*, where *Q *is well approximated by Equations (11) and (12). Instead, they used the expression,

Q⌣≈πR2c/c02,     (23)
 MathType@MTEF@5@5@+=feaafiart1ev1aaatCvAUfKttLearuWrP9MDH5MBPbIqV92AaeXatLxBI9gBaebbnrfifHhDYfgasaacH8akY=wiFfYdH8Gipec8Eeeu0xXdbba9frFj0=OqFfea0dXdd9vqai=hGuQ8kuc9pgc9s8qqaq=dirpe0xb9q8qiLsFr0=vr0=vr0dc8meaabaqaciaacaGaaeqabaqabeGadaaakeaacuWGrbqugaafaiabgIKi7IGaciab=b8aWjabdkfasnaaCaaaleqabaGaeGOmaidaaOGaem4yamMaei4la8Iaem4yam2aa0baaSqaaiabicdaWaqaaiabikdaYaaakiabcYcaSiaaxMaacaWLjaWaaeWaceaacqaIYaGmcqaIZaWmaiaawIcacaGLPaaaaaa@3EED@

where *c*_0 _is the Korteweg-Moens wave velocity for a perfect liquid in an elastic tube, [(*Eh*)/(2*ρR*)]^1/2^. In their reviews of the West et al. model, Dodds et al. [14] and Chaui-Blinkerd [15] also assumed that pulsatile flow is described by Equation (23). The constant *E *is the elastic modulus of the wall describing radial tension. Because *h/R *is nearly the same in a parent artery and in the daughter arteries, *c*_0 _is nearly equal in arteries connected at a branching.

West et al. used the relationship *c*^2^/c02
 MathType@MTEF@5@5@+=feaafiart1ev1aaatCvAUfKttLearuWrP9MDH5MBPbIqV92AaeXatLxBI9gBaebbnrfifHhDYfgasaacH8akY=wiFfYdH8Gipec8Eeeu0xXdbba9frFj0=OqFfea0dXdd9vqai=hGuQ8kuc9pgc9s8qqaq=dirpe0xb9q8qiLsFr0=vr0=vr0dc8meaabaqaciaacaGaaeqabaqabeGadaaakeaacqWGJbWydaqhaaWcbaGaeGimaadabaGaeGOmaidaaaaa@3008@ ≈ -*J*_2_(*i*^3/2^*αR*)/*J*_0_(*i*^3/2^*αR*) to estimate blood flow. For *αR *>> 1, -*J*_2_(*i*^3/2^*αR*)/*J*_0_(*i*^3/2^*αR*) ≈ 1, and blood flow rate computed from Equation (23) is proportional to *R*^2^. Assuming that the energy cost of pumping blood through arteries is entirely the cost of the oscillating flow, the energy minimization principle used by Murray and West et al. leads to the conclusion that the exponent in the equation Rkλ=Rk+1,1λ+Rk+1,2λ+⋯+Rk+1,ηλ
 MathType@MTEF@5@5@+=feaafiart1ev1aaatCvAUfKttLearuWrP9MDH5MBPbIqV92AaeXatLxBI9gBaebbnrfifHhDYfgasaacH8akY=wiFfYdH8Gipec8Eeeu0xXdbba9frFj0=OqFfea0dXdd9vqai=hGuQ8kuc9pgc9s8qqaq=dirpe0xb9q8qiLsFr0=vr0=vr0dc8meaabaqaciaacaGaaeqabaqabeGadaaakeaacqWGsbGudaqhaaWcbaGaem4AaSgabaacciGae83UdWgaaOGaeyypa0JaemOuai1aa0baaSqaaiabdUgaRjabgUcaRiabigdaXiabcYcaSiabigdaXaqaaiab=T7aSbaakiabgUcaRiabdkfasnaaDaaaleaacqWGRbWAcqGHRaWkcqaIXaqmcqGGSaalcqaIYaGmaeaacqWF7oaBaaGccqGHRaWkcqWIVlctcqGHRaWkcqWGsbGudaqhaaWcbaGaem4AaSMaey4kaSIaeGymaeJaeiilaWIae83TdGgabaGae83UdWgaaaaa@4FAF@ is approximately 2 when *αR *>> 1. However, for *αR *< 1, -*J*_2_(*i*^3/2^*αR*)/*J*_0_(*i*^3/2^*αR*) ≈ *i*(*αR*)^2^/8, and Equation (23) leads to the conclusion that *Q *is proportional to *R*^3 ^(again assuming that *h/R *is invariant). However, this prediction contradicts the prediction of Equation (12) that *Q *is approximately proportional to *R*^4 ^for *αR *< 1.

Another error in the argument of West et al. results from their reliance on complex-variable valued expressions for pressure gradient and blood flow. For an oscillating pressure gradient with period 2*π*/*ω*, the rate of work per unit length required to pump blood over the time cycle from -*π*/*ω *to *π*/*ω *can be calculated by integrating the product of the expression for the pressure gradient and the expression for blood flow. However, the result of this calculation is incorrect if the integration is performed using complex-variable solutions for these expressions. This can be demonstrated for the case of the oscillating pressure gradient *Ăe*^*iωt*^: the integral of the pressure gradient multiplied by flow rate is 0 when pressure and flow are the complex-variable solutions. However, heat is produced during the cycle by shear forces in the liquid. Clearly, this contradicts the laws of thermodynamics. A correct calculation of the average rate of energy dissipation can be made from the product of the real part of the solution for the pressure gradient and the real part of the solution for the blood flow equation.

The real part of the oscillatory pressure gradient is

Re⁡{ΔP⌣/L}=A⌣(eiωt+e−iωt)/2.     (24)
 MathType@MTEF@5@5@+=feaafiart1ev1aaatCvAUfKttLearuWrP9MDH5MBPbIqV92AaeXatLxBI9gBaebbnrfifHhDYfgasaacH8akY=wiFfYdH8Gipec8Eeeu0xXdbba9frFj0=OqFfea0dXdd9vqai=hGuQ8kuc9pgc9s8qqaq=dirpe0xb9q8qiLsFr0=vr0=vr0dc8meaabaqaciaacaGaaeqabaqabeGadaaakeaacyGGsbGucqGGLbqzcqGG7bWEcqGHuoarcuWGqbaugaafaiabc+caViabdYeamjabc2ha9jabg2da9iqbdgeabzaauaWaaeWaceaacqWGLbqzdaahaaWcbeqaaiabdMgaPHGaciab=L8a3jabdsha0baakiabgUcaRiabdwgaLnaaCaaaleqabaGaeyOeI0IaemyAaKMae8xYdCNaemiDaqhaaaGccaGLOaGaayzkaaGaei4la8IaeGOmaiJaeiOla4IaaCzcaiaaxMaadaqadiqaaiabikdaYiabisda0aGaayjkaiaawMcaaaaa@501F@

The solution for the rate of energy dissipation (per unit length) that is attributable to the oscillatory component of blood flow is equal to the real part of the product of Expression (24) and the expression for the rate of blood flow, Equation (12). The integral with respect to time of this product from -*π*/*ω *to *π*/*ω *divided by 2*π*/*ω *gives the average rate of energy dissipation (per unit length) resulting from the oscillatory component, W⌣/L=π(A⌣2/2)R4/(8μ)eiθPQ−iθJ0|PQ(i3/2αR)|/|J0(i3/2αR)|
 MathType@MTEF@5@5@+=feaafiart1ev1aaatCvAUfKttLearuWrP9MDH5MBPbIqV92AaeXatLxBI9gBaebbnrfifHhDYfgasaacH8akY=wiFfYdH8Gipec8Eeeu0xXdbba9frFj0=OqFfea0dXdd9vqai=hGuQ8kuc9pgc9s8qqaq=dirpe0xb9q8qiLsFr0=vr0=vr0dc8meaabaqaciaacaGaaeqabaqabeGadaaakeaacuWGxbWvgaafaiabc+caViabdYeamjabg2da9GGaciab=b8aWnaabmGabaGafmyqaeKbaqbadaahaaWcbeqaaiabikdaYaaakiabc+caViabikdaYaGaayjkaiaawMcaaiabdkfasnaaCaaaleqabaGaeGinaqdaaOGaei4la8YaaeWaceaacqaI4aaocqWF8oqBaiaawIcacaGLPaaacqWGLbqzdaahaaWcbeqaaiabdMgaPjab=H7aXnaaBaaameaacqWGqbaucqWGrbquaeqaaSGaeyOeI0IaemyAaKMae8hUde3aaSbaaWqaaiabdQeakjabicdaWaqabaaaaOWaaqWaceaacqWGqbaudaWgaaWcbaGaemyuaefabeaakmaabmGabaGaemyAaK2aaWbaaSqabeaacqaIZaWmcqGGVaWlcqaIYaGmaaGccqWFXoqycqWGsbGuaiaawIcacaGLPaaaaiaawEa7caGLiWoacqGGVaWldaabdiqaaiabdQeaknaaBaaaleaacqaIWaamaeqaaOWaaeWaceaacqWGPbqAdaahaaWcbeqaaiabiodaZiabc+caViabikdaYaaakiab=f7aHjabdkfasbGaayjkaiaawMcaaaGaay5bSlaawIa7aaaa@6AA7@, which has the real part

Re⁡{W⌣/L}=π(A⌣2/2)R4/(8μ)cos⁡(−θPQ+θJ0)|PQ(i3/2αR)|/|J0(i3/2αR)|.     (25)
 MathType@MTEF@5@5@+=feaafiart1ev1aaatCvAUfKttLearuWrP9MDH5MBPbIqV92AaeXatLxBI9gBaebbnrfifHhDYfgasaacH8akY=wiFfYdH8Gipec8Eeeu0xXdbba9frFj0=OqFfea0dXdd9vqai=hGuQ8kuc9pgc9s8qqaq=dirpe0xb9q8qiLsFr0=vr0=vr0dc8meaabaqaciaacaGaaeqabaqabeGadaaakeaacyGGsbGucqGGLbqzcqGG7bWEcuWGxbWvgaafaiabc+caViabdYeamjabc2ha9jabg2da9GGaciab=b8aWnaabmGabaGafmyqaeKbaqbadaahaaWcbeqaaiabikdaYaaakiabc+caViabikdaYaGaayjkaiaawMcaaiabdkfasnaaCaaaleqabaGaeGinaqdaaOGaei4la8YaaeWaceaacqaI4aaocqWF8oqBaiaawIcacaGLPaaacyGGJbWycqGGVbWBcqGGZbWCdaqadiqaaiabgkHiTiab=H7aXnaaBaaaleaacqWGqbaucqWGrbquaeqaaOGaey4kaSIae8hUde3aaSbaaSqaaiabdQeakjabicdaWaqabaaakiaawIcacaGLPaaadaabdiqaaiabdcfaqnaaBaaaleaacqWGrbquaeqaaOWaaeWaceaacqWGPbqAdaahaaWcbeqaaiabiodaZiabc+caViabikdaYaaakiab=f7aHjabdkfasbGaayjkaiaawMcaaaGaay5bSlaawIa7aiabc+caVmaaemGabaGaemOsaO0aaSbaaSqaaiabicdaWaqabaGcdaqadiqaaiabdMgaPnaaCaaaleqabaGaeG4mamJaei4la8IaeGOmaidaaOGae8xSdeMaemOuaifacaGLOaGaayzkaaaacaGLhWUaayjcSdGaeiOla4IaaCzcaiaaxMaadaqadiqaaiabikdaYiabiwda1aGaayjkaiaawMcaaaaa@781C@

It is clear from Table 1 that when *αR *≤ 1 the angle *θ*_*d *_= -*θ*_*PQ *_+ *θ*_*J*0 _is small and cos(*θ*_*d*_) ≈ 1. Furthermore, when *αR *≤ 1, the quantity |*P*_*Q*_(*i*^3/2^*αR*)|/|*J*_0_(*i*^3/2^*αR*)| is close to 1. Consequently, for *αR *≤ 1, we have Re{W⌣
 MathType@MTEF@5@5@+=feaafiart1ev1aaatCvAUfKttLearuWrP9MDH5MBPbIqV92AaeXatLxBI9gBaebbnrfifHhDYfgasaacH8akY=wiFfYdH8Gipec8Eeeu0xXdbba9frFj0=OqFfea0dXdd9vqai=hGuQ8kuc9pgc9s8qqaq=dirpe0xb9q8qiLsFr0=vr0=vr0dc8meaabaqaciaacaGaaeqabaqabeGadaaakeaacuWGxbWvgaafaaaa@2DFE@/*L*} ≈ *π*(*Ă*^2^/2)*R*^4^/(8*μ*). Therefore, energy dissipation for the oscillatory component is proportional to *R*^4^. The rate of energy dissipation per unit length for the constant, forward-pumping component of flow is easily shown to be *πÃ*^2^*R*^4^/(8*μ*). Therefore, as previously stated without formal proof by Bengtsson and Edén [16], the total rate of energy dissipation in this case is proportional to *R*^4^. Consequently, Murray's optimization procedure leads to Murray's Law when *αR *≤ 1.

To evaluate energy dissipation due to the oscillatory component of pressure when *αR *> 1, we start by rewriting Equation (11) as

Q⌣=πR2A⌣eiωt−iπ/2+iθJ2−iθJ0/(ωρ)|J2(i3/2αR)|/|J0(i3/2αR)|.
 MathType@MTEF@5@5@+=feaafiart1ev1aaatCvAUfKttLearuWrP9MDH5MBPbIqV92AaeXatLxBI9gBaebbnrfifHhDYfgasaacH8akY=wiFfYdH8Gipec8Eeeu0xXdbba9frFj0=OqFfea0dXdd9vqai=hGuQ8kuc9pgc9s8qqaq=dirpe0xb9q8qiLsFr0=vr0=vr0dc8meaabaqaciaacaGaaeqabaqabeGadaaakeaacuWGrbqugaafaiabg2da9GGaciab=b8aWjabdkfasnaaCaaaleqabaGaeGOmaidaaOGafmyqaeKbaqbacqWGLbqzdaahaaWcbeqaaiabdMgaPjab=L8a3jabdsha0jabgkHiTiabdMgaPjab=b8aWjabc+caViabikdaYiabgUcaRiabdMgaPjab=H7aXnaaBaaameaacqWGkbGscqaIYaGmaeqaaSGaeyOeI0IaemyAaKMae8hUde3aaSbaaWqaaiabdQeakjabicdaWaqabaaaaOGaei4la8YaaeWaceaacqWFjpWDcqWFbpGCaiaawIcacaGLPaaadaabdiqaaiabdQeaknaaBaaaleaacqaIYaGmaeqaaOWaaeWaceaacqWGPbqAdaahaaWcbeqaaiabiodaZiabc+caViabikdaYaaakiab=f7aHjabdkfasbGaayjkaiaawMcaaaGaay5bSlaawIa7aiabc+caVmaaemGabaGaemOsaO0aaSbaaSqaaiabicdaWaqabaGcdaqadiqaaiabdMgaPnaaCaaaleqabaGaeG4mamJaei4la8IaeGOmaidaaOGae8xSdeMaemOuaifacaGLOaGaayzkaaaacaGLhWUaayjcSdGaeiOla4caaa@7080@

The integral defining the average rate of energy dissipation (per unit length) that is attributable to oscillatory pressure is

*πR*^2^*Ă*^2 ^cos(-*π*/2 + *θ*_*J*2 _- *θ*_*J*0_)/(2*ωρ*)|*J*_2_(*i*^3/2^*αR*)|/|*J*_0_(*i*^3/2^*αR*)|.

For *αR *>> 1, |*J*_2_(*i*^3/2^*αR*)|/|*J*_0_(*i*^3/2^*αR*)| approaches 1, and *θ*_*J*2 _- *θ*_*J*0 _approaches *π *[17]. Consequently, cos(-*π*/2 + *θ*_*J*2 _- *θ*_*J*0_) goes to 0, and the above rate of energy dissipation is not proportional to *R*^2 ^as claimed by West et al. When energy dissipation that is attributable to the oscillating pressure gradient is very small compared with energy dissipation resulting from the constant, forward-pumping component of the gradient, Murray's procedure again leads to the conclusion that Murray's Law is approximately correct.

## Discussion

The exact solution for blood velocity in the tethered elastic tube model in this paper does not contain terms with a frequency greater than the frequency *ω *of the oscillating pressure gradient. In contrast, the approximate solutions of Womersley [6] contain exponential functions with frequencies that are integral multiples of *ω*. If the terms with a frequency greater than *ω *resulted from one or more of the approximations, then the results of Womersley do not provide a valid explanation for the reversal of flow during early systole in large blood vessels, e.g., the reversal shown in Figure 5 of the article by Womersley [6].

While Womersley did include a term describing an elastic tethering force in his last publication [18], he did not reach an exact solution for the modified model. The exact solution for the tethered elastic tube model provides the basis for demonstrating that the solutions for blood flow and shear force in the rigid tube are good approximations for the oscillatory component of blood flow and shear force, respectively, in arteries. These approximations are used to calculate shear stress at the inner wall of an artery as a plausible explanation for the scaling described in Murray's Law. The same approximations lead to the conclusion that blood flow and energy dissipation are not proportional to *R*^2 ^in large arteries. This result leads to the conclusion that the "general model for the origin of allometric scaling laws in biology" of West et al. [13] does not support a 3/4-power scaling law for mammalian BMR.

Since Murray published his explanation for the scaling of the radii of blood vessels, a number of investigations have shown that the exponent *λ *in the relationship, Rkλ=Rk+1,1λ+Rk+1,2λ+⋯+Rk+1,ηλ
 MathType@MTEF@5@5@+=feaafiart1ev1aaatCvAUfKttLearuWrP9MDH5MBPbIqV92AaeXatLxBI9gBaebbnrfifHhDYfgasaacH8akY=wiFfYdH8Gipec8Eeeu0xXdbba9frFj0=OqFfea0dXdd9vqai=hGuQ8kuc9pgc9s8qqaq=dirpe0xb9q8qiLsFr0=vr0=vr0dc8meaabaqaciaacaGaaeqabaqabeGadaaakeaacqWGsbGudaqhaaWcbaGaem4AaSgabaacciGae83UdWgaaOGaeyypa0JaemOuai1aa0baaSqaaiabdUgaRjabgUcaRiabigdaXiabcYcaSiabigdaXaqaaiab=T7aSbaakiabgUcaRiabdkfasnaaDaaaleaacqWGRbWAcqGHRaWkcqaIXaqmcqGGSaalcqaIYaGmaeaacqWF7oaBaaGccqGHRaWkcqWIVlctcqGHRaWkcqWGsbGudaqhaaWcbaGaem4AaSMaey4kaSIaeGymaeJaeiilaWIae83TdGgabaGae83UdWgaaaaa@4FAF@, is close to 3 for mammalian arteries [19-22]. While the mechanism responsible for this scaling of vessel radius is a matter of some speculation, the SFR hypothesis is an attractive explanation for the scaling. As shown previously by Kassab and Fung for constant pressure gradients [5] and in this paper for pulsatile gradients, the constant shear force assumption leads to the conclusion that arterial radii follow Murray's Law for all but the largest arteries.

As reviewed by Barakat et al. [4], a causal role for shear stress in determining the radius of an artery is supported by experimental observations. However, much of the evidence that supports the SFR hypothesis also supports the cost minimization hypothesis of Murray. Whether Murray's hypothesis, the SFR hypothesis or some other proposal is the correct explanation for the scaling of the radii of arteries ultimately depends on biological plausibility, and this will largely depend on observations from experimental studies.

## Competing interests

The author(s) declare that they have no competing interests.

## Authors' contributions

The authors contributed equally to the calculation of bounds on *D *and the calculation of energy dissipation for oscillatory flow. The tethered elastic tube model, the exact solution for flow and shear force and the relation between shear force and Murray's law were contributed by PP.

## Abbreviations

BMR Basal metabolic rate

SFR Shear force remodeling

*R *Radius of an artery measured from the central axis to the inner wall

*r *Distance from the central axis of an artery to a point in the arterial bloodstream

*η *Number of daughter arteries connected to the parent artery at a branching

*h *Thickness of the arterial wall

*Z *Displacement of the inner arterial wall caused by oscillatory shear force and measured parallel to the central axis

*ν*_*w *_Velocity of the inner arterial wall measured parallel to the central axis

*u *Velocity of arterial blood measured parallel to the central axis: *ŭ *denotes the velocity for a harmonic pressure gradient with mean 0, and *ũ *denotes the velocity for a constant pressure gradient.

*A *Pressure gradient at a point in an artery: an oscillating gradient with mean equal to 0 is denoted *Ă*, and a constant gradient is denoted *Ã*.

*Q *Rate of blood flow measured as volume per second: Q⌣
 MathType@MTEF@5@5@+=feaafiart1ev1aaatCvAUfKttLearuWrP9MDH5MBPbIqV92AaeXatLxBI9gBaebbnrfifHhDYfgasaacH8akY=wiFfYdH8Gipec8Eeeu0xXdbba9frFj0=OqFfea0dXdd9vqai=hGuQ8kuc9pgc9s8qqaq=dirpe0xb9q8qiLsFr0=vr0=vr0dc8meaabaqaciaacaGaaeqabaqabeGadaaakeaacuWGrbqugaafaaaa@2DF2@ denotes the rate for an oscillating pressure gradient with mean 0, and Q˜
 MathType@MTEF@5@5@+=feaafiart1ev1aaatCvAUfKttLearuWrP9MDH5MBPbIqV92AaeXatLxBI9gBaebbnrfifHhDYfgasaacH8akY=wiFfYdH8Gipec8Eeeu0xXdbba9frFj0=OqFfea0dXdd9vqai=hGuQ8kuc9pgc9s8qqaq=dirpe0xb9q8qiLsFr0=vr0=vr0dc8meaabaqaciaacaGaaeqabaqabeGadaaakeaacuWGrbqugaacaaaa@2DE6@ denotes the rate for a constant pressure gradient.

W⌣
 MathType@MTEF@5@5@+=feaafiart1ev1aaatCvAUfKttLearuWrP9MDH5MBPbIqV92AaeXatLxBI9gBaebbnrfifHhDYfgasaacH8akY=wiFfYdH8Gipec8Eeeu0xXdbba9frFj0=OqFfea0dXdd9vqai=hGuQ8kuc9pgc9s8qqaq=dirpe0xb9q8qiLsFr0=vr0=vr0dc8meaabaqaciaacaGaaeqabaqabeGadaaakeaacuWGxbWvgaafaaaa@2DFE@/*L *Rate of energy dissipation per unit length that is attributable to the oscillatory component of blood flow.

*ω *Frequency of an oscillating pressure gradient in radians per second

*ρ *Density of blood

*μ *Viscosity of blood

*α *(*ωρ*/*μ*)^1/2^

*K *Coefficient of arterial wall elastic deformation resulting from shear force

κ Kh

*J*_*m*_(*i*^3/2^*αR*) Bessel function of order *m *and variable *i*^3/2^*αR*, ∑n=0n=∞(−1)n(i3/2αR/2)m+2n/[(m+n)!n!]
 MathType@MTEF@5@5@+=feaafiart1ev1aaatCvAUfKttLearuWrP9MDH5MBPbIqV92AaeXatLxBI9gBaebbnrfifHhDYfgasaacH8akY=wiFfYdH8Gipec8Eeeu0xXdbba9frFj0=OqFfea0dXdd9vqai=hGuQ8kuc9pgc9s8qqaq=dirpe0xb9q8qiLsFr0=vr0=vr0dc8meaabaqaciaacaGaaeqabaqabeGadaaakeaadaaeWaqaamaabmGabaGaeyOeI0IaeGymaedacaGLOaGaayzkaaWaaWbaaSqabeaacqWGUbGBaaGcdaqadiqaaiabdMgaPnaaCaaaleqabaGaeG4mamJaei4la8IaeGOmaidaaGGacOGae8xSdeMaemOuaiLaei4la8IaeGOmaidacaGLOaGaayzkaaaaleaacqWGUbGBcqGH9aqpcqaIWaamaeaacqWGUbGBcqGH9aqpcqGHEisPa0GaeyyeIuoakmaaCaaaleqabaGaemyBa0Maey4kaSIaeGOmaiJaemOBa4gaaOGaei4la8YaamWaceaadaqadiqaaiabd2gaTjabgUcaRiabd6gaUbGaayjkaiaawMcaaiabcgcaHiabd6gaUjabcgcaHaGaay5waiaaw2faaaaa@5560@

*P*_*Q*_(*i*^3/2^*αR*) [*R*^2^*i*^3/2^*αJ*_0_(*i*^3/2^*αR*) - 2*RJ*_1_(*i*^3/2^*αR*)]/(*R*^4^/8)

*P*_*S*_(*i*^3/2^*αR*) *J*_1_(*i*^3/2^*αR*)/[(*R*/2)(*i*^3/2^*α*)]

*θ*_*J*0 _Argument of *J*_0_(*i*^3/2^*αR*)

*θ*_*PQ *_Argument of *P*_*Q*_(*i*^3/2^*αR*)

*θ*_*PS *_Argument of *P*_*S*_(*i*^3/2^*αR*)

*θ*_*d *_-*θ*_*J*0 _+ *θ*_*PQ*_
